# In vitro comparison of primary stability of two implant designs in D3 bone

**DOI:** 10.4317/medoral.21714

**Published:** 2017-06-04

**Authors:** José González-Serrano, Ricardo Ortega-Aranegui, Juan López-Quiles

**Affiliations:** 1 Student of Master’s Programme in Oral Surgery and Implantology. Department of Oral Medicine and Surgery, School of Dentistry, Complutense University, Madrid, Spain; 2 Associate Professor. Department of Oral Medicine and Surgery, School of Dentistry, Complutense University, Madrid, Spain; 3 Director of Master’s Programme in Oral Surgery and Implantology. Department of Oral Medicine and Surgery, School of Dentistry, Complutense University, Madrid, Spain

## Abstract

**Background:**

Primary stability (PS) is a key factor for implant survival rate and depends on implant design or bone quality. The aim of this study was to compare different thread designs implants, evaluating PS with periotest values (PV) and implant stability quotient (ISQ) values through resonance frequency analysis (RFA).

**Material and Methods:**

A total of 60 implants (Radhex®, Inmet-Garnick S.A., Guadalajara, Spain) were placed in freshly bovine ribs *in vitro*. Two designs were used: 30 tapered body with single thread design (PHI) and 30 tapered body with double thread design implants (PHIA). Both designs were 4mm wide and 12mm long. Implants were placed according to manufacturer’s guidelines. Osstell™ and Periotest® devices were used to evaluate PS by a blinded independent observer. Computed tomographies (CTs) of the ribs were made (BrightSpeed Series CT systems, GE Healthcare, Milwaukee, WI, USA) and bone quality surrounding each implant was evaluated in Hounsfield Units (HU) using Ez3D Plus software (Vatech Co., Korea). Bone quality was classified according to Misch and Kircos in D1, D2, D3 or D4.

**Results:**

All implants were mechanically stable. Only implants placed in D3 bone (350-850 HU) were selected for the study: 28 PHI and 26 PHIA. The one way ANOVA showed significant difference (*p*<0.005) among two implants designs in ISQ values (61,55 ± 6,67 in PHI and 68,94 ± 5,82 in PHIA). No significant difference (*p* = 0,171) was shown in PV between two designs (-4,47 ± 1,39 in PHI and -4,77 ± 0,87 in PHIA).

**Conclusions:**

Higher PS was found using Osstell™ device in implants with double thread design (PHIA) in comparison to implants with single thread design (PHI) in D3 bone.

** Key words:**Dental implant, primary stability, Osstell, Periotest, bone quality, implant design.

## Introduction

Primary stability (PS) of dental implants is a decisive factor for its success. PS depends on length, diameter, shape and thread design of the implant, the insertion technique or the type of bone (1). However, there are no established measurement standards (2).

There is enough evidence to confirm that an increase in bone quality also increase the PS of the implant (3,4). Implants placed in low quality bone, those contacting only in cancellous bone, as found in the posterior maxilla, seems to have higher failure rates (5). According to Misch and Kircos (6) and Lekholm and Zarb (7) classifications, low quality bone corresponds to bone type 3 and 4.

Implant design play a key role in order to obtain good bone to implant contact and particularly when immediate loading is needed (8). Several studies reported higher PS in parallel implants when compared to tapered ones (9,10), while other studies found the opposite (11,12). Rokn *et al.* (12) also found implant length not to have significant differences in PS but an increase in implant diameter to have an increase in PS. In addition, pitch distance of the screw threads is not clear to be important in achieving better PS (13,14).

For these reasons, manufacturers are in continuous research into implant design in order to improve PS in low quality bone (15,16). Radhex® (Inmet-Garnick, S.A., Guadalajara, Spain) uses a subtractive surface treatment by shot blasting and incorporates a double thread implant design which they ensure to improve PS. Nevertheless, there is no study evaluating PS of these implants. Therefore, the aim of this study was to determine *in vitro* the PS of different Radhex® dental implant designs in low quality bone.

## Material and Methods

**Figure 1:**
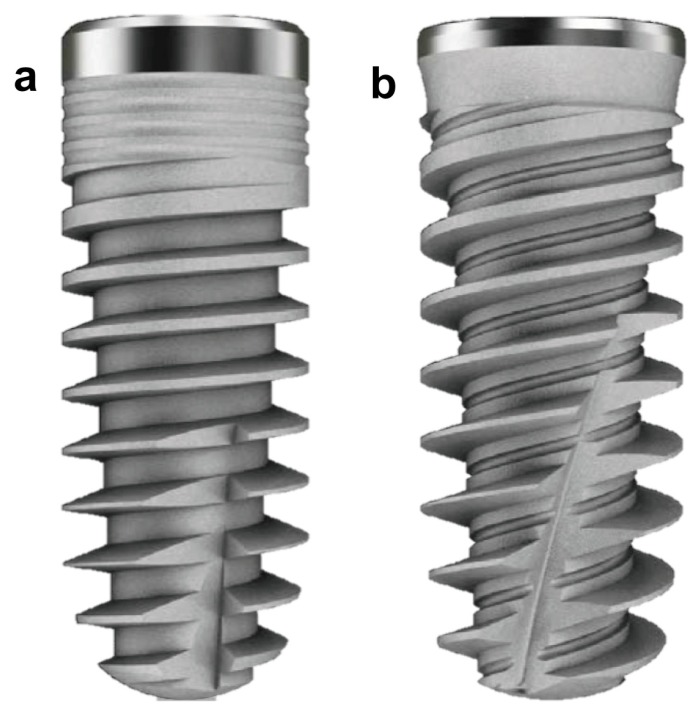
Different Radhex® implant designs used in this study: (a) PHI and (b) PHIA.

Thirty fresh bovine ribs were randomly selected after the complete removal of the soft tissues. Two different dental implants were chosen to be compared: tapered body with single thread design implant (PHI) (Fig. 1a) and tapered body with double thread design implant (PHIA) (Fig. 1b). Both designs were 4 mm wide and 12 mm long. Thirty PHI and 30 PHIA implants were inserted with 40 Ncm or less in 30 bovine ribs, placing both designs in each rib.

- PS measurements

PS measurements were assessed by an oral surgeon blinded to the study protocol. Wireless resonance frequency analysis (RFA) device (Osstell AB, Gothenburg, Sweden) and wireless electronic percussive test (Periotest M, Medizintechnik Gulden, Modautal, Germany) were used.

**Figure 2:**
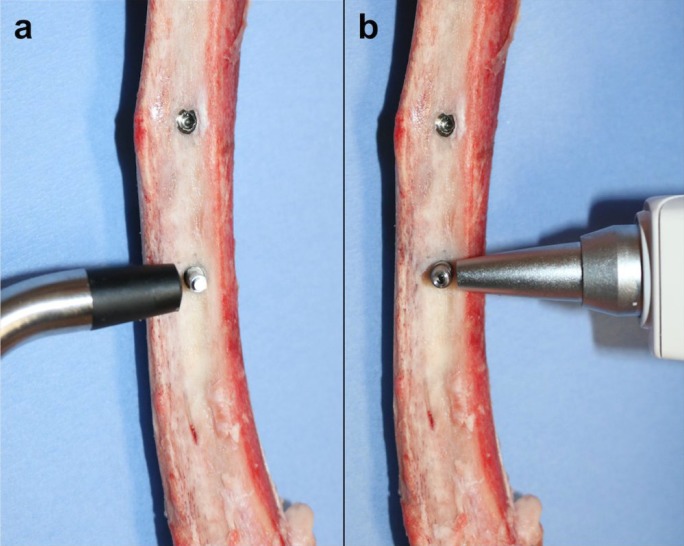
Evaluation of PS using (a) Osstell™ and (b) Periotest® devices.

Firstly, implant stability quotient (ISQ) was measured using RFA device. A suitable-transducter was inserted into the implant body (Smart Peg). Measurements were done in two different directions of the implant, perpendicular to the Smart Peg according to manufacturer guidelines (Fig. 2a).

After ISQ values were obtained from all implants, abutments were placed and periotest values (PV) were evaluated in every implant. The final PV of each implant was obtained after three consecutive measurements, taking the average value as the final PV of each implant (Fig. 2b).

-Bone quality assessment

**Figure 3:**
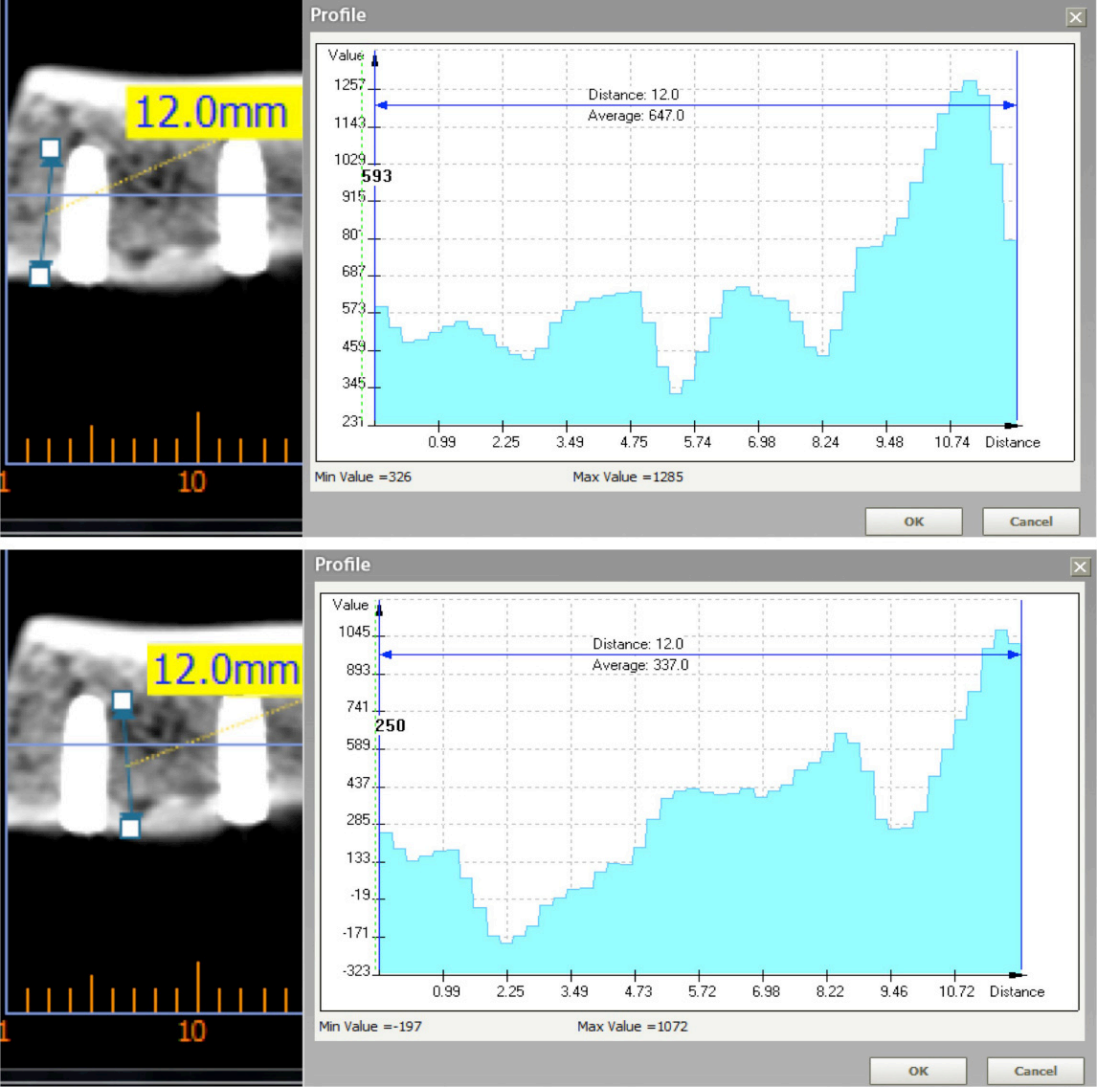
Panoramic section of a CT scan. Bone quality average recorded in HU of the implant recipient area measured on each side of it using Ez3D plus software.

After placing the implants, six computed tomographies (CTs) of 5 ribs each were made (BrightSpeed Series CT systems, GE Healthcare, Milwaukee, WI, USA). Hounsfield units (HU) were evaluated in each rib using Ez3D Plus software for Windows (Vatech Co., Korea). Panoramic sections of each rib were made in order to calculate bone quality. Two measures (in each side of the implant) were made, taking as final result the mean value between them (Fig. 3).

Bone quality from each implant was classified in HU according to Misch and Kircos (6) ( Table 1). Only implants placed in D3 bone (350-850 HU) were selected in this study (54 implants out of 60: 28 PHI and 26 PHIA).

**Table 1:**
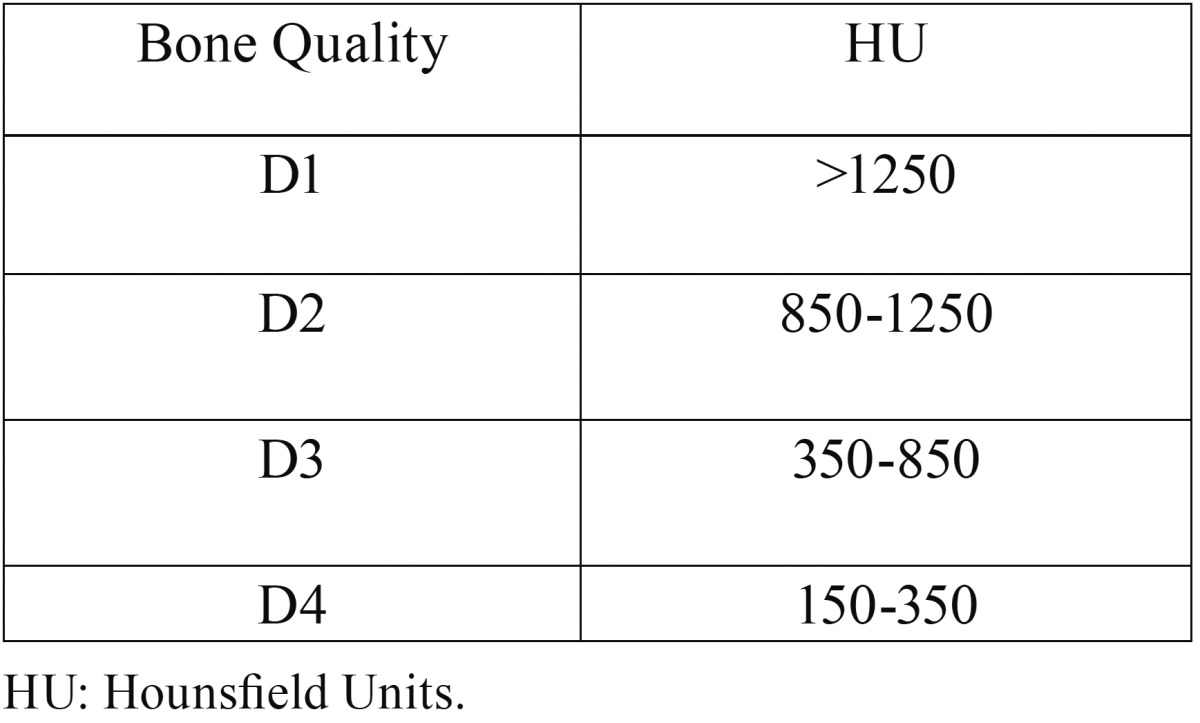
Bone classification according to Misch and Kircos.

- Statistical Analysis

Statistical software SPSS for Windows version 20 (SPSS Inc, Chicago, IL, USA) was used for the statistical analysis. Descriptive statistics (means and standard deviations) were applied for ISQ, PV and bone quality of each implant design. One-way analysis of variance (ANOVA) was used for statistical evaluation. In order to quantify the concordance between measurements assessed with Osstell™ and Periotest devices of PHI and PHIA groups, an intraclass correlation coefficient (ICC) model two-ways, mixed effects was performed. The results were assessed with 95% confidence intervals at a significance level of *p*<.05.

## Results

All implants were mechanically stable. No mobility was observed. The mean ISQ in PHI implants was 61.55 ± 6.67 and 68.94 ± 5.82 in PHIA group. The mean PV was -4.47 ± 1.39 and -4.77 ± 0.87 respectively ( Table 2).

**Table 2:**
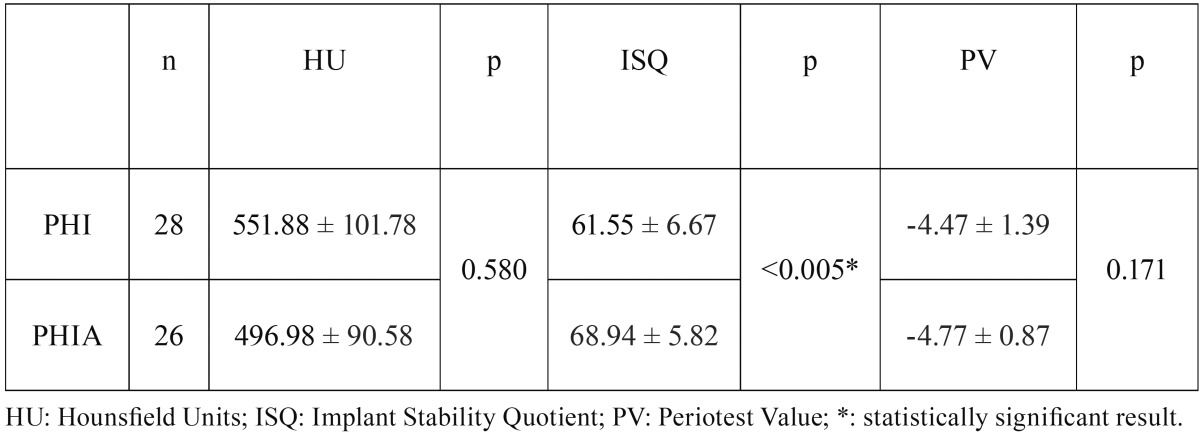
Comparison of bone quality in relation to HU and PS according to ISQ and PV between the different implant designs studied.

The one way ANOVA showed statistically significant difference among two implants designs in ISQ (*p*<0.005). No statistically significant difference was shown in PV between two designs (*p*=0.171).

The ICC between Osstell™ and Periotest devices was r=0.26 for PHI design and r=0.004 for PHIA group.

## Discussion

Today, several ways of implant preparation have been described. Synthetic bones, cadaveric bone, resin models or animal bone are the most frequent (17). Artificial bone can reproduce different bone densities and report thermal conductivity (18). In our study, as animal bone was used, bone quality was assessed in HU with Ez3D viewer according to Misch and Kircos classification. This software has been recently used to compare condylar morphologies (19). Herekar *et al.* (20) also used CT data to classify bone quality according to Misch and Kircos classification.

The final location of the implants was not randomized. This could have affected the type of bone surrounding each implant, as the most proximal sector of the bovine ribs present a higher ratio of cancellous to cortical bone (21), which may have influenced the PS results. However, bone quality of each implant group was not statistically significant (551.88 ± 101.78 HU in PHI and 496.98 ± 90.58 HU in PHIA, p=0.580).

In the present study, Osstell™ and Periotest® were used to assess PS measurements. Osstell™ works transferring vibration frequencies onto an implant transducer, which generates a value in ISQ units. RFA is an accepted method, as it is objective and reliable (22). Periotest® measures PS by assessing the damping capacity upon tapping against the implant and generating a PV. It has also been shown to be a reliable indicator in conventional and immediate loading (23). However, Osstell™ system has been proved to be more reliable compared to Periotest® in measuring dental implant stability (24).

Although higher bone quality was observed surrounding PHI implants, higher ISQ and better PV in PHIA were obtained. PHIA dental implants showed statistically higher ISQ values in D3 bone compared to PHI implants. Also, better PV of PHIA compared to PHI implants was observed, but no statistically significant differences were found. These results are similar to those obtained in the study by Park *et al.* (25). They obtained statistically significant differences in ISQ but not in PV when comparing two different implant designs. This can be explained due to an average range of -5 to +5 PV reported in the literature (26), which is a small range for implant stability and makes more difficult to achieve significant differences in the evaluation (27).

The two implant designs tested in this study present a similar body (both are tapered), however, the differences between thread designs (PHI has single thread design and PHIA has double thread design) may have been the main reason for these results. This study proved that double thread design implants (PHIA) had better PS in comparison to single thread design implants (PHI). The reason may have been a better bone to implant contact obtained in trabecular bone with PHIA implants.

ISQ value of PHI implants was 61.55 ± 6.67, which allow us to perform a conventional loading (28). On the other hand, ISQ value of PHIA implants was 68.94 ± 5.82, which would allow us to perform an early loading (29). Therefore, PHIA implants seems to be more suitable in low quality bones. PV of PHI implants was -4.47 ± 1.39 and -4.77 ± 0.87 for PHIA design, which indicates a good integration, meaning that implant loading could be performed according to the manufacturer.

As an experimental study carried out on bovine ribs, it was not possible to simulate *in vivo* conditions such as the access to the surgical site or the blood supply to the bone. Hence, although preliminary data is important, further clinical studies are needed to confirm these findings. Within the limitations of this study, we can conclude that higher implant PS was found using Osstell™ device in tapered body and double thread design implants (PHIA) in comparison to tapered body and single thread design im-plants (PHI) in D3 bone.
